# In Vitro Propagation of *Humulus lupulus* through the Induction of Axillary Bud Development

**DOI:** 10.3390/plants11081066

**Published:** 2022-04-13

**Authors:** Takeshi Hirakawa, Seia Tanno

**Affiliations:** Kirin Central Research Institute, Kirin Holdings Company, Ltd., 26-1, Muraoka-Higashi 2, Fujisawa 251-8555, Kanagawa, Japan; seia_tanno@kirin.co.jp

**Keywords:** propagation, shoot branching, gibberellic acid, cytokinin

## Abstract

*Humulus lupulus* (hop) is a necessary material for beer brewing. Improved breeding cultivars of hops with enhanced tolerance to environmental stresses, such as drought and heat stress, accompanying climate change have been developed. However, a propagation system, which is needed for the proliferation of new cultivars, is not currently available for hops. In this study, we found that treatment of stem explants with 0.01–0.05 ppm gibberellic acid (GA_3_) induced the development of axillary buds in the hop cultivar Kirin-2, resulting in the proliferation of shoot branching. Additionally, 0.01 ppm benzyl adenine (BA) enhanced the development of axillary buds formed in response to 0.05 ppm GA_3_ in various hop cultivars, particularly Nugget. The development of axillary buds was strongly repressed by the application of 0.05 ppm BA at a concentration equal to the 0.05 ppm GA_3_ concentration, which showed the possibility that a high concentration of cytokinin preferentially prevents the effect of GA_3_ on the development of axillary buds in hops. These results indicated that combined treatment of stem explants with GA_3_ and cytokinin at appropriate concentrations is effective for the propagation of proliferated hop cultivars through shoot branching.

## 1. Introduction

*Humulus lupulus* var. *lupulus* (hop) is a perennial, dioecious, and climbing plant belonging to the Cannabaceae family. Hop is widely cultivated in areas under a microthermal climate worldwide and is used as a key ingredient in beer brewing because it brings a floral aroma, bitterness, and foam stability [1].

The female flower of the hop called the “hop cone” contains a unique structure and function. In hop cones, the base of bracts surrounded with strig and bracteoles possess glandular trichomes known as lupulin grands, which secrete cells and biosynthesize various kinds of bioactive secondary metabolites, such as prenylated flavonoids (xanthohumol and desmethylxanthohumol), bitter acids (humulone (α-acid) and lupulone (β-acid)) and essential oils [2]. The secondary metabolites derived from the hop cone benefit not only beer brewing but also animal and human diseases. For example, xanthohumol shows multifunctional anticancer activity on various organs of the body [3]. Additionally, iso-α-acids, which are hop-derived bitter acids in beer, suppress amyloid β deposition in a mouse model of Alzheimer’s disease, suggesting that iso-α-acids prevent dementia [4,5].

The demand for high-quality hops has increased with the diversification of the brewing industry and preferences for beer. In addition, it is expected that the quality and yield of hops will decrease in the production area due to global warming [6,7,8]. To solve this problem, improved cultivars of hops have been developed using classical intercrosses to increase their quality and yield in warm areas. Because hops are propagated vegetatively, the rapid production or proliferation of new cultivars requires many plantlets but not seeds. The basic methods for the tissue culture of hops are applicable in various cultivars, but there are few reports on the propagation system of hops [9,10]. In propagation systems of some species of plants, the induction of shoot branching based on the development of axillary buds is effective in increasing the numbers of shoot apexes and stem nodes, which are used as materials for plantlets [11,12,13]. In this study, we present a method for inducing the development of axillary buds in hop cultivars Kirin-2, Cascade, and Nugget. We found that the formation of axillary buds in stem explants was promoted by gibberellic acid (GA_3_). Additionally, the application of cytokinin enhanced the elongation of axillary buds formed with GA_3_, which indicated that the combination treatment of GA_3_ and cytokinin is useful for the proliferation of hops through the induction of shoot branching.

## 2. Results

### 2.1. Glucose Is Suitable as a Sugar Source in the Axillary Bud Development of Hop

**Figure 1:**
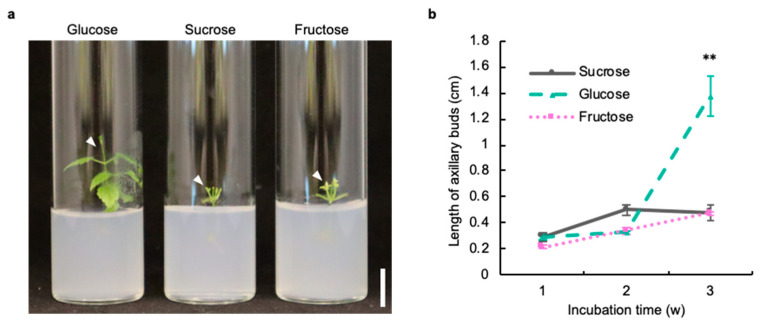
The development of axillary buds of hop requires glucose as a sugar source. (**a**) Axillary bud development in stem explants of hop cultivar Kirin-2 cells were treated with glucose, sucrose, or fructose for 3 weeks. The white arrowheads indicate elongated axillary buds. Scale bar: 1 cm. (**b**) Length of axillary buds in stem explants treated with glucose, sucrose, or fructose each week for 3 weeks (*n* ≥ 10 individual experiments, ** *p* < 0.01; Tukey–Kramer test).

Sugar plays an important role in regulating growth and development in plant tissue culture. In the tissue culture of hops, different types of sugar are used as a sugar source by each hop cultivar [9,10,14]. Thus, we checked the response of hops to glucose, sucrose, and fructose with respect to the axillary bud development of stem explants using the hop cultivar Kirin-2, which is an improved cultivar of hop cv. Shinshu Wase in Japan. The hop cultivar Kirin-2 shows a higher yield and larger amounts of α- and β-acids than the hop cultivar Shinshu Wase. At 3 weeks after incubation, the length of axillary buds of stem explants treated with glucose was 3 times longer than that of stem explants treated with glucose and fructose (glucose: 1.4 cm, sucrose: 0.48 cm, fructose: 0.47 cm) (Figure 1a,b). This result suggested that glucose was most suitable as a sugar for the induction of axillary bud development in the hop cultivar Kirin-2.

### 2.2. Cytokinin Inhibits the Development of Axillary Buds in Hop

**Figure 2:**
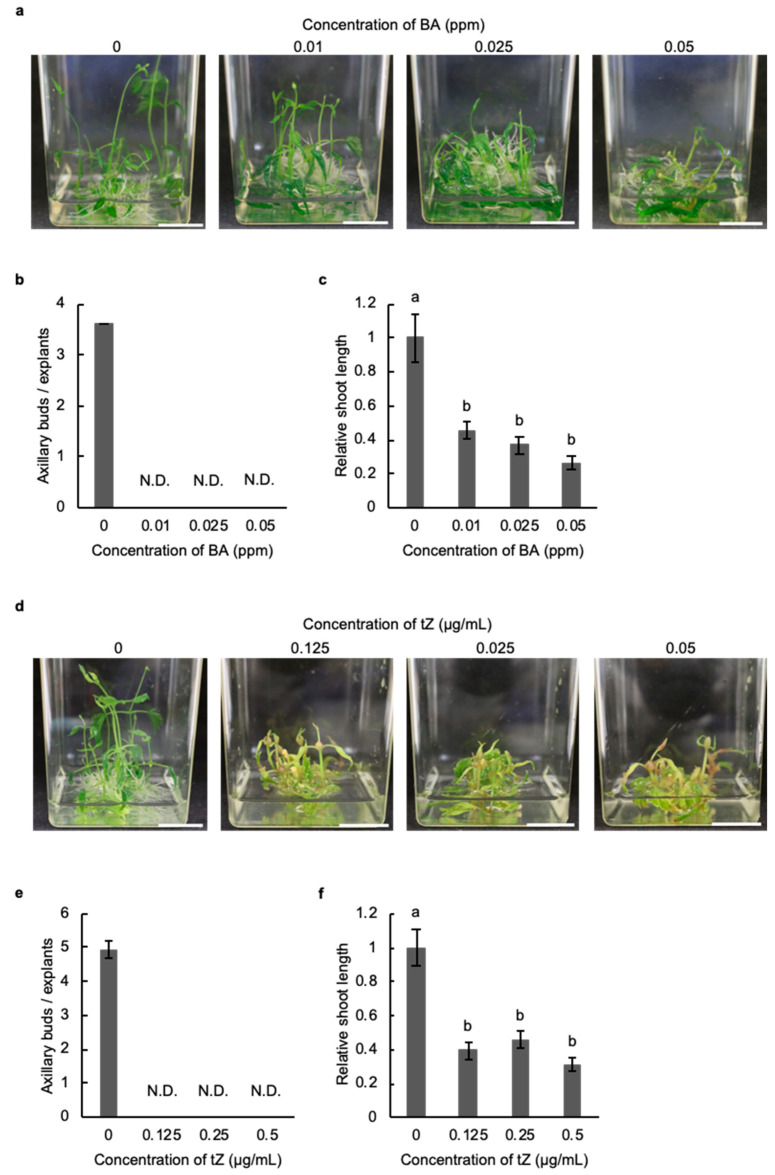
Cytokinin inhibits the development of axillary buds in hop. (**a**) Axillary bud development in stem explants of the hop cultivar Kirin-2 in liquid medium supplemented with benzyl adenine (BA) for 3 weeks. Scale bar: 1 cm. (**b**) The number of axillary buds in stem explants without and with BA for 3 weeks. N.D. indicates no detection (*n* ≥ 15, individual experiment). (**c**) Relative shoot length in stem explants treated without and with BA for 3 weeks. The shoot length of explants treated with BA was normalized against the shoot length of explants without BA (*n* ≥ 10, individual experiment, *p* < 0.05; Tukey–Kramer test). Different letters indicate significant differences. (**d**) Axillary bud development in stem explants of the hop cultivar Kirin-2 in liquid medium supplemented with *trans*-zeatin (tZ) for 3 weeks. Scale bar: 1 cm. (**e**) Number of axillary buds in stem explants without and with tZ for 3 weeks. N.D. indicates no detection (*n* ≥ 15, individual experiment). (**f**) Relative shoot length of stem explants without and treated with tZ for 3 weeks. The shoot length of explants treated with tZ was normalized against the shoot length of explants without tZ (*n* ≥ 10, individual experiment, *p* < 0.05; Tukey–Kramer test). Different letters indicate significant differences.

Cytokinin induces axillary bud development in a wide range of plant species [15]. To investigate the effect of cytokinin on the development of axillary buds in hop, we observed sensitivity to benzyl adenine (BA) or *trans*-zeatin (tZ) in stem explants of the hop cultivar Kirin-2. Under normal conditions, axillary buds developed from stem explants in liquid culture (Figure 2a,b,d,e). In contrast, the application of BA or tZ inhibited the formation of axillary buds in stem explants (Figure 2b,e). The length of shoots in stem explants treated with BA and tZ was 2.5 times shorter than that of stem explants without BA and tZ (Figure 2c,d,f). Additionally, the discolouration of explants was induced by tZ treatment (Figure 2d). These results suggested that cytokinin inhibited the formation of axillary buds and shoot development in hop.

### 2.3. Induction of Axillary Bud Formation with Gibberellic Acid

**Figure 3:**
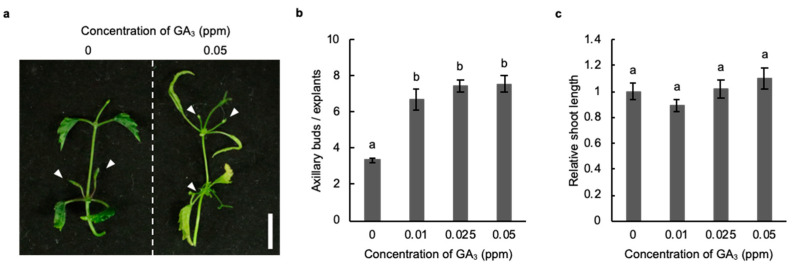
The development of axillary buds was induced by gibberellic acid (GA_3_) in hop. (**a**) Stem explants of the hop cultivar Kirin-2 treated without and with 0.05 ppm GA_3_ for 3 weeks. The white arrowheads indicate the axillary buds. Scale bar: 1 cm. (**b**) Number of axillary buds in stem explants without and with GA_3_ for 3 weeks (*n* ≥ 15, individual experiment, *p* < 0.05; Tukey–Kramer test). Different letters indicate significant differences. (**c**) Relative shoot length of stem explants treated without and with GA_3_. The shoot length of explants treated with GA_3_ was normalized against the shoot length of explants without GA_3_ for 3 weeks (*n* = 10, individual experiment, no significant difference; Tukey–Kramer test). Different letters indicate significant differences.

In several species of plants, GA_3_ treatment induces the development of axillary buds [16,17]. Thus, we examined whether the development of axillary buds was induced with GA_3_ in hop cv. Kirin-2. Under normal conditions, axillary buds were not confirmed at stem nodes near the shoot apex (Figure 3a). However, the formation of axillary buds was observed at stem nodes near the shoot apex in explants treated with GA_3_. The number of axillary buds in stem explants treated with GA_3_ was twice as large as that in stem explants without GA_3_ (Figure 3b). GA_3_ treatment did not induce internode elongation in the main stem explants, leading to the GA_3_-induced promotion of axillary bud formation in hops (Figure 3c).

### 2.4. Combined Treatment with Gibberellic Acid and Cytokinin Strongly Promotes the Development of Axillary Buds

**Figure 4:**
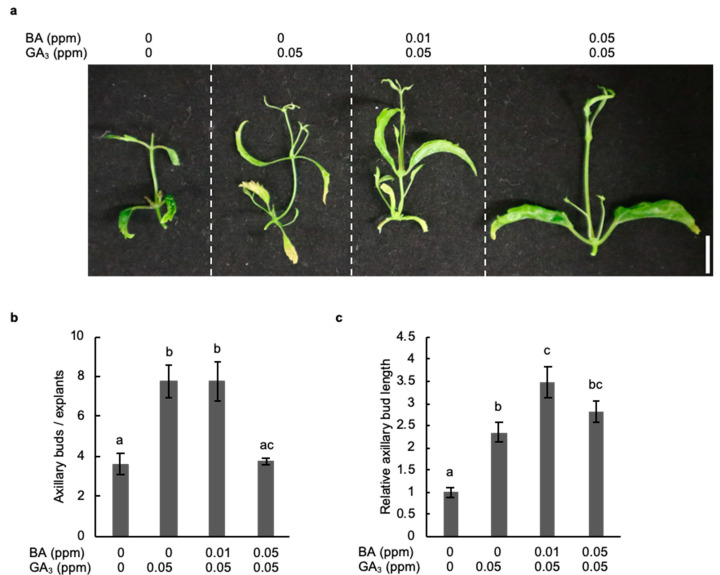
Combination treatment with GA_3_ and BA strongly induces the development of axillary buds in hop. (**a**) Stem explants of hop cultivar Kirin-2 cells were treated with GA_3_ and BA for 3 weeks. Scale bar: 1 cm. (**b**) Number of axillary buds in stem explants with GA_3_ and BA for 3 weeks (*n* ≥ 14, individual experiment, *p* < 0.05; Tukey–Kramer test). Different letters indicate significant differences. (**c**) Relative length of axillary buds of stem explants treated without and with GA_3_ and BA for 3 weeks. The axillary bud length of explants treated with GA_3_ and BA was normalized against the axillary bud length of explants without GA_3_ and BA for 3 weeks (*n* ≥ 22, individual experiment, *p* < 0.05; Tukey–Kramer test). Different letters indicate significant differences.

To verify that BA enhances the effect of GA_3_ on axillary bud development in hop cv. Kirin-2, we observed the development of axillary buds in stem explants treated with both GA_3_ and BA. The detection frequency of axillary buds in stem explants treated with both GA_3_ and BA was comparable with that in stem explants treated with only GA_3_ (Figure 4a,b). In addition, combined treatment with GA_3_ and BA markedly increased the length of axillary buds by 1.7 times compared with that obtained with the GA_3_ treatment (Figure 4c). These results suggested that cytokinin enhanced the elongation of axillary buds formed by GA_3_ treatment in hop.

**Figure 5:**
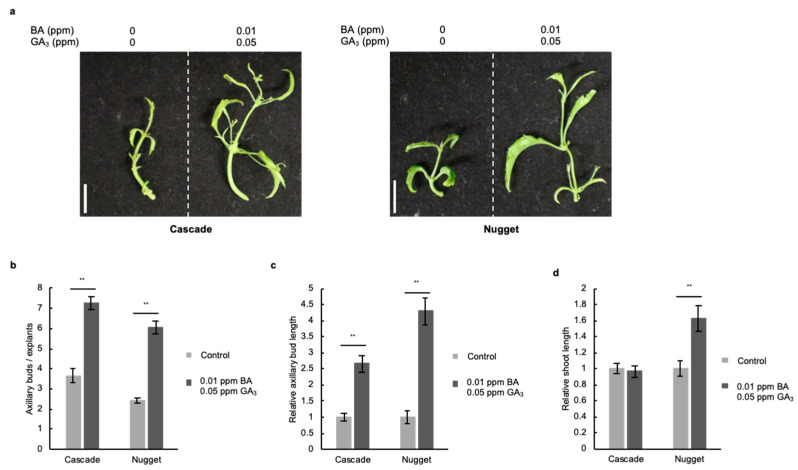
The development of axillary buds is enhanced with gibberellic acid (GA_3_) and BA in various hop cultivars. (**a**) Stem explants of the hop cultivars Cascade and Nugget were treated with 0.01 ppm BA and 0.05 ppm GA_3_ for 3 weeks. Scale bars: 1 cm. (**b**) Number of axillary buds in stem explants treated with 0.01 ppm BA and 0.05 ppm GA_3_ for 3 weeks (*n* ≥ 9, individual experiment, *p* < 0.01; Student’s *t* test). (**c**) Relative axillary bud length of stem explants treated without and with 0.01 ppm BA and 0.05 ppm GA_3_ for 3 weeks. The axillary bud length of explants treated with 0.01 ppm BA and 0.05 ppm GA_3_ was normalized against the axillary bud length of explants without BA and GA_3_ (*n* ≥ 12, individual experiment, ** *p* < 0.01; Student’s *t* test). (**d**) Relative shoot length of stem explants treated without and with 0.01 ppm BA and 0.05 ppm GA_3_ for 3 weeks. The shoot length of explants treated with 0.01 ppm BA and 0.05 ppm GA_3_ was normalized against the shoot length of explants without BA and GA_3_ (*n* ≥ 10, individual experiment, ** *p* < 0.01; Student’s *t* test).

We then investigated the effect of GA_3_ and cytokinin on axillary bud development in hop cultivars other than Kirin-2 using Cascade and Nugget, which are mainly cultivated in the US and Germany and show resistance to downy resistance [18,19]. Cascade shows a low amount of α-acid and an aroma with medium strength. Additionally, Nugget is the variety which has high α-acid and mild aroma. The combined treatment with BA and GA_3_ increased the number of axillary buds in stem explants of Cascade and Nugget by 2 and 2.5 times, respectively, compared with that without BA and GA_3_ (Figure 5a,b). The length of axillary buds in stem explants of Cascade and Nugget treated with BA and GA_3_ was 2.7 and 4.3 times, respectively, higher than that of stem explants without BA and GA_3_ (Figure 5c). In addition, the shoot length in stem explants in Nugget but not in Cascade increased 1.6 times after the GA_3_ and BA treatments, which indicated that GA_3_ and cytokinin might have the ability to enhance the development of axillary buds in various hop cultivars, unlike the results obtained for shoot development (Figure 5d).

### 2.5. Rooting and Transplanting in Hop

**Figure 6:**
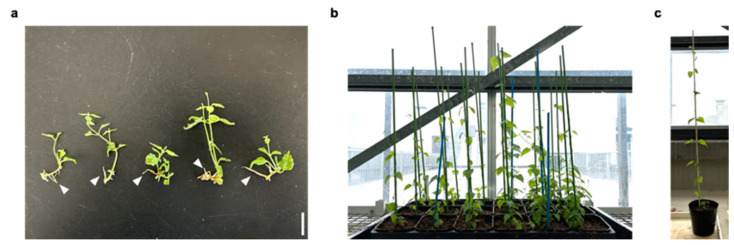
Rooting and transplanting of hop. (**a**) Stem explants with roots of the hop cultivar Kirin-2 at 2 weeks after the induction of rooting with 0.05 ppm 1-Naphthaleneacetic acid. The white arrowheads indicate the roots. Scale bar: 1 cm. (**b**) Plantlets of the hop cultivar Kirin-2 at 1.5 months after transplanting. (**c**) Plantlets of the hop cultivar Kirin-2 at 2 months after transplanting.

Finally, we checked whether the induction of axillary buds was useful in the plantlet propagation of hops. The rooting of stem explants derived from propagated plants through the induction of axillary buds development was observed with 1-Naphthaleneacetic acid (NAA) treatment (Figure 6a). Additionally, transplanting of stem explants with roots was confirmed in a greenhouse under natural photoperiod conditions, suggesting that our findings might help increase the efficiency of plantlet propagation of hops (Figure 6b,c).

## 3. Discussion

In this study, our sugar application test first revealed that glucose is most suitable as a sugar source for the induction of axillary bud development in stem explants of the hop cultivar Kirin-2 (Figure 1a,b). Different types of sugar were used as a sugar source in tissue culture of each hop cultivar in previous studies. For example, sucrose is used as a sugar source in the hop cultivars Hallertauer Mitterlfrüeh, Columbus, and Chinook [14]. In contrast, glucose is supplemented in the growth medium of the hop cultivars Cascade and H138 [9,10]. Thus, it might be important to select a suitable sugar source for the induction of axillary bud development in each hop cultivar.

We revealed that combined treatment with GA_3_ and BA induces the development of axillary buds in stem explants of hop. The molecular mechanism underlying the development of axillary buds has been studied for several decades using model plants [20]. In *Arabidopsis thaliana* (*A. thaliana*), *BRANCHED1/2* (*BRC1/2*), which belongs to the TCP transcription factor family and is related to *TEOSINTE BRACNCHED1* (*TB1*) in *Zea mays* and *FINE CULM1* (*FC1*) in *Oryza sativa*, is a negative regulator of axillary bud development [21,22,23]. The expression of *BRC1/2* is activated by increasing the concentration of auxin transported from the shoot apex in the main stem through PIN-FORMED (PIN) auxin-efflux facilitators [21]. In addition, high levels of cytokinin reduced the expression of *BRC1/2*, resulting in induction of axillary bud development, which suggested that auxin derived from the shoot apex inhibits axillary bud development through the repression of cytokinin biosynthesis at stem nodes. Recent studies showed that the homologue of *BRC1/2* also plays an important role in the development of axillary buds in the climbing plant *Cucumber sativus* [24]. Cucumber *BRANCHED1* (*CsBRC1*) reduces the expression levels of *PIN1/3* through direct interaction with their promoter regions, and knockdown mutants of *CsBRC1* exhibit overshoot branching [25].

Although cytokinin induces the development of axillary buds in *Cannabis sativa*, which belongs to the Cannabaceae family, as well as in most species of plants, our phytohormone application test showed that axillary buds developed with GA_3_ but not BA in hops [26]. GA_3_ inhibits the outgrowth of axillary buds through regulation of the auxin levels and transport in model plants such as *A. thaliana* and *Populus* [27,28]. In contrast, the development of axillary buds is promoted by GA_3_ in *Phaseolus vulgaris*, citrus, and snapdragon, as well as in hops, which suggests that the application of GA_3_ might be useful for the induction of axillary buds in plant species that are insensitive to cytokinin application [12,13]. The combined treatment with BA and GA_3_ induces the development of axillary buds in stem explants to a greater degree than in plants treated with only GA_3_ in woody plants, such as *Jatropha curcas* (*J. curcas*) [29]. Although BA enhanced the elongation of axillary buds induced with GA_3_ in stem explants of hops as well as *J. curcas*, the formation of axillary buds was strongly inhibited by the application of BA at a concentration equal to the GA_3_ concentration (Figure 4a). These results show the possibility that a high concentration of cytokinin preferentially prevents the effect of GA_3_ on the induction of axillary buds in hops.

Previous studies have shown that GA_3_ is involved in the development of hop cones in various cultivars. GA_3_ application increases the number of hop cones with changes in the α-acid contents in the hop cultivars Saazer, Hüller Anfang, and Hallertauer in the field compared with the variation in the number of hop cones observed with GA_3_ in the cultivar Fuggle [30,31]. Our phytohormone application tests also showed that the sensitivity to GA_3_ of shoot development differs between the hop cultivars Cascade and Nugget (Figure 5d). These findings suggest that the molecular mechanisms controlling growth and development involving GA_3_ are complicated in hop cultivars. Recently, high-quality draft genomes have been constructed through sequencing and *de novo* assembly in some hop cultivars [32,33]. This information might contribute to revealing the molecular mechanism underlying growth and development in the presence of GA_3_.

## 4. Materials and Methods

### 4.1. Plant Materials and Growth Conditions

*Humulus lupulus* cv. Kirin-2, Cascade, and Nugget were used as materials in this study. To obtain the in vitro plants, stem explants were isolated from seedlings and sterilized with 70% (*w*/*v*) ethanol and 1% (*w*/*v*) sodium hypochlorite for 1 min and 5 min, respectively. After being washed 3 times with sterilized water, the stem explants were transferred to 1/2 Murashige and Skoog (MS) medium supplemented with 2% (*w*/*v*) glucose, 0.01 ppm benzyl adenine (BA), and 0.8% (*w*/*v*) agar at pH 5.8. The samples were moved into an incubator set at 20 °C with a 16 h light/8 h dark photoperiod. After 3 weeks, developed axillary buds with stem explants were transferred to 1/2 MS medium supplemented with 2% glucose and 0.8% agar to establish the in vitro plants in hormone-free conditions. The in vitro plants were subcultured every 1.5 months.

### 4.2. Sugar Application Test

Stem explants isolated from 1.5-month-old in vitro plants were incubated in 1/2 MS medium supplemented with 0.8% agar and 2% glucose, sucrose, or fructose. At each week for 3 weeks, the length of axillary buds was measured. At least two independent experiments were performed, and similar results were obtained.

### 4.3. Phytohormone Application Test

Stem explants isolated from 1.5-month-old in vitro plants were incubated in 1/2 MS liquid medium supplemented with 2% glucose, BA, *trans*-zeatin (Tokyo Chemical Industry, Tokyo, Japan), and GA_3_ (FUJIFILM Wako, Japan). After 3 weeks, the shoot length, axillary bud length and number of axillary buds of the explants were measured using ImageJ software. To calculate the relative shoot and axillary bud lengths, the shoot and axillary bud lengths of explants treated with each phytohormone were normalized against the shoot and axillary bud lengths of explants without phytohormones. At least two independent experiments were performed, and similar results were obtained.

### 4.4. Rooting and Transplanting

Stem explants isolated from 1-month-old propagated plants through the induction of axillary bud development were incubated on papers containing 1/2 MS medium supplemented with 2% glucose and 0.05 ppm NAA (FUJIFILM Wako, Japan) in an incubator at 20 °C. After 2 weeks, stem explants with roots were transferred to the cell trays with soil consisting of Metro-Mix 350 (Sun Gro Horticulture, Agawam, MA, USA), vermiculite, and red ball earth (=3:1:1). Plantlets were grown in a greenhouse (25 °C) under natural photoperiod conditions.

### 4.5. Statistical Analysis

Statistical analysis was performed using Student’s *t* test and the Tukey–Kramer test. The sample size and *p* value are presented in the Figure Legends.

## Data Availability

The data presented in this study are available on reasonable request from the corresponding author.

## Abbreviations

Hop	*Humulus lupulus* var. *lupulus*
GA_3_	Gibberellic acid
BA	Benzyl adenine
tZ	*trans*-Zeatin
*BRC1/2*	*BRACNHED1/2*
*TB1*	*TEOSINTE BRACNCHED1*
*FC1*	*FINE CULM1*
*PIN*	*PIN-FORMED*
NAA	1-Naphthaleneacetic acid
